# The Role of MUC1 in Renal Cell Carcinoma

**DOI:** 10.3390/biom14030315

**Published:** 2024-03-07

**Authors:** Martina Milella, Monica Rutigliano, Francesco Lasorsa, Matteo Ferro, Roberto Bianchi, Giuseppe Fallara, Felice Crocetto, Savio Domenico Pandolfo, Biagio Barone, Antonio d’Amati, Marco Spilotros, Michele Battaglia, Pasquale Ditonno, Giuseppe Lucarelli

**Affiliations:** 1 Urology, Andrology and Kidney Transplantation Unit, Department of Precision and Regenerative Medicine and Ionian Area, University of Bari “Aldo Moro”, 70124 Bari, Italypasquale.ditonno@uniba.it (P.D.); 2 Division of Urology, European Institute of Oncology, Istituto di Ricovero e Cura a Carattere Scientifico (IRCCS), 71013 Milan, Italy; 3 Department of Neurosciences, Science of Reproduction and Odontostomatology, University of Naples Federico II, 80131 Naples, Italy; 4 Department of Urology, University of L’Aquila, 67010 L’Aquila, Italy; 5 Division of Urology, Department of Surgical Sciences, AORN Sant’Anna e San Sebastiano, 81100 Caserta, Italy; 6 Pathology Unit, Department of Precision and Regenerative Medicine and Ionian Area, University of Bari “Aldo Moro”, 70124 Bari, Italy

**Keywords:** mucin-1, MUC1, cancers, renal cell carcinoma, CA15-3, therapy

## Abstract

Mucins are a family of high-molecular-weight glycoproteins. MUC1 is widely studied for its role in distinct types of cancers. In many human epithelial malignancies, MUC1 is frequently overexpressed, and its intracellular activities are crucial for cell biology. MUC1 overexpression can enhance cancer cell proliferation by modulating cell metabolism. When epithelial cells lose their tight connections, due to the loss of polarity, the mucins become dispersed on both sides of the epithelial membrane, leading to an abnormal mucin interactome with the membrane. Tumor-related MUC1 exhibits certain features, such as loss of apical localization and aberrant glycosylation that might cause the formation of tumor-related antigen epitopes. Renal cell carcinoma (RCC) accounts for approximately 3% of adult malignancies and it is the most common kidney cancer. The exact role of MUC1 in this tumor is unknown. Evidence suggests that it may play a role in several oncogenic pathways, including proliferation, metabolic reprogramming, chemoresistance, and angiogenesis. The purpose of this review is to explore the role of MUC1 and the meaning of its overexpression in epithelial tumors and in particular in RCC.

## 1. Introduction

Mucins are a family of high-molecular-weight glycoproteins. Among them, transmembrane protein mucin-1 (MUC1) is widely studied for its role in several kinds of cancers [1]. Usually, epithelial cells’ surfaces are covered in MUC1, which creates a dense mesh that protects cells from harsh external circumstances, forming a protective barrier across the mucosal surface [2]. In many epithelial malignancies, including breast, colon, liver, lung, ovarian, and pancreatic cancer, MUC1 is frequently overexpressed, and its intracellular activities are crucial for cell biology. Indeed, this protein takes part in several signaling pathways involved in cell growth, proliferation, metabolic reprogramming, apoptosis, and developmental processes [3,4]. Therefore, MUC1 might be utilized in clinical settings as a diagnostic marker for cancer development and recurrence and might be even used as a therapeutic target. The aim of this review is to explore the role of MUC1 and the meaning of its overexpression in epithelial tumors and in particular in renal cell carcinoma (RCC).

## 2. MUC1 Structure and Biological Properties

Epithelial tissues are made up of apical–basal polarized cells that are linked laterally. Due to their location, they are largely exposed to environmental factors, requiring sophisticated and well-developed defense mechanisms to keep them intact. Mucins accumulate on the surface of the epithelial cells and are involved in their survival, repair, and defense [5]. MUC1 consists of two subunits: the transmembrane carboxy-terminal subunit (MUC1–C) and the extracellular amino-terminal subunit (MUC1–N). Within the GSVV motif in the sea urchin protein enterokinase and agrin (SEA) domain, this single polypeptide chain is autoproteolytically cleaved into two fragments: the longer N-terminal subunit and the shorter C-terminal subunit. This cleavage happens immediately after translation and is caused by conformational stress [6]. Stable hydrogen bonds keep the two subunits connected extracellularly. The highly preserved variable number tandem repeats (VNTRs) of 20 amino acids found in the extracellular MUC1-N are rich in serine (Ser), threonine (Thr), and proline (Pro) residues. The N-terminus is drastically modified by *O*-linked glycans (Thr and Ser residues) [7,8,9]. Notably, the MUC1-N region acts as a cell barrier, preventing interactions between cells and the extracellular matrix as well as protecting them from pathogenic and cellular invasions while maintaining and repairing the epithelium. Sometimes, MUC1-N is released from the cell surface, leaving MUC1-C as a possible receptor. This putative receptor can be phosphorylated and take part in a variety of signaling cascades linked to transformation and tumor growth [10]. Because of its position, the C-terminus of MUC1 has been the subject of increasing research, particularly in relation to inflammation and cancer development. MUC1-C has a cytoplasmic tail (72 amino acidic residues), a transmembrane structural domain (28 amino acids), and an extracellular domain (58 amino acids) [11]. Moreover, MUC1-C accumulates in the cytosol and then targets the cell’s nucleus and mitochondria [12]. Crucially, transformation and resistance to stress-driven apoptosis can only be produced by overexpressing MUC1-C and not the MUC1-N component [13,14,15,16]. The direct binding and stabilization of β-catenin by MUC1-C facilitates the activation of Wnt target genes [17]. In response to genotoxic stressors, MUC1-C can directly interact with p53 in the nucleus to activate the p21 gene [18].

## 3. MUC1 Function and Its Influence on Metabolism

Significant glycosylation of the MUC1 extracellular subunit is present in normal tissues to lubricate the underlying epithelia while shielding the tissues [19]. As a result, proteolytic cleavage can release the N-terminal component into the extracellular environment. It is shed off the cell surface, creating a barrier against invasive infections. Several inflammatory components, including TNF-α and IFN-γ, may induce these protective responses. Some enzymes, including metalloproteinase 17 and TNF-α-converting enzyme, also mediate this process [20,21]. Moreover, protein kinase C (PKC), glycogen synthase kinase 3 (GSK3), tyrosine-protein kinase Abl, non-receptor tyrosine kinase (Src), EGFR, tyrosine-protein kinase MET, and glycogen synthase kinase 3β can all phosphorylate MUC1-C [22]. MUC1 controls the flow of metabolites and acts as a transcriptional coactivator, directly controlling the expression of genes involved in metabolism. Moreover, it regulates the signaling of receptor tyrosine kinase (RTK), which contributes to producing the biosynthetic intermediates needed for cell development. Furthermore, MUC1 promotes cancer cell survival in hypoxic and nutrient-deprived environments by regulating autophagy, reactive oxygen species levels, and metabolite flux [23]. MUC1 interacts with two important transcription factors that directly control the expression of metabolic genes: p53 and hypoxia-inducible factor-1 alpha (HIF-1α) [24]. In particular, MUC1 directly regulates the expression of glycolytic genes (including HK2, PFKFB2, ENO1, PGK1, PGM2, LDHA, and GLUT1) and promotes increased glucose uptake and utilization in cancer cells in a HIF-dependent manner [24]. In addition, MUC1 interacts with HIF-1α, regulating the stability and activation of this transcription factor, and it is itself a target of HIF-1α [25]. Thus, a positive feedback loop would be established between MUC1 and HIF-1α that may serve as a global metabolic regulator in cancer cells.

**Figure 1:**
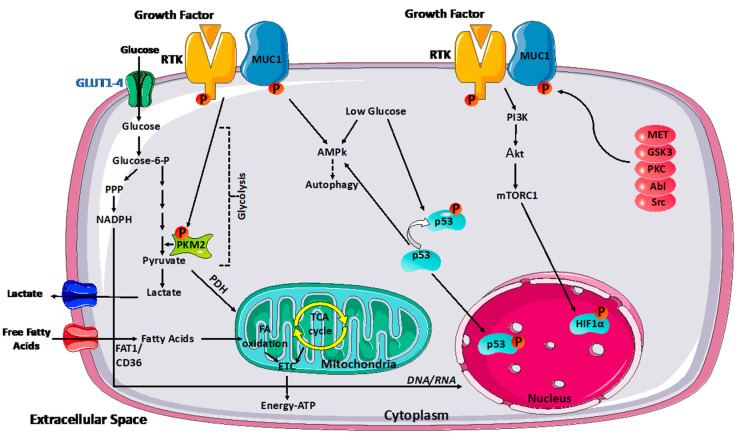
MUC1 modulation of cancer metabolism. MUC1 modulates glycolytic flow by activating various signaling pathways, including PI3K/Akt/mTOR, p53, and HIF-1α, influencing glycolytic gene transcription. Under glucose-depleted circumstances, MUC1 activates AMPK, which supports survival by inducing autophagy. MUC1-RTK interaction enhances RTK membrane stability. As a result, the complex enhances downstream signaling of RTK, which can affect PKM2 activity and transcriptional activation of other glycolytic genes. Reciprocally, RTKs phosphorylate MUC1 and facilitate signaling downstream of MUC1. MUC1 also interacts directly with glycolytic enzymes such as PKM2 to control carbon flux. Moreover, protein kinase C (PKC), glycogen synthase kinase 3 (GSK3), tyrosine–protein kinase Abl, non-receptor tyrosine kinase (Src), and tyrosine–protein kinase MET can all phosphorylate MUC1.

The tricarboxylic acid cycle (TCA), the pentose phosphate pathway (PPP), and fatty acid synthesis are all affected by changes in MUC1 expression. The PPP leads to the production of NADPH, which is required for anabolic processes of cell metabolism. The PPP results in ribose production which is essential for de novo DNA and RNA synthesis, which is crucial for tumor cell proliferation. In addition to its role as a transcriptional co-activator, MUC1 also directly controls the activities of metabolic enzymes to manage the flux of carbon [26]. Therefore, MUC1 overexpression can enhance cancer cell proliferation by modulating cell metabolism (Figure 1).

## 4. MUC1: Pro- or Anti-Inflammatory Role?

MUC1 has an established capacity to respond to inflammation by activating healing-associated responses with proliferation and remodeling. As a consequence of this protective role and the increasing evidence of chronic inflammation, prolonged MUC1 activation drives multiple hallmarks of the cancer cell, such as the epithelial–mesenchymal transition (EMT), epigenetic reprogramming, chromatin remodeling, stemness, and pluripotency factor expression. By functioning as an immunomodulatory switch, MUC1 can have a pro- or anti-inflammatory effect in different diseases. In tumors, MUC1 can interact directly with dendritic cells (DCs) and macrophages in a pro-inflammatory manner. It can regulate the recruitment of inflammatory cells, encourage cancer immune evasion, and establish a distinct inflammatory cell milieu inside the tumor microenvironment [27]. It suppresses the DC response, which is necessary for inflammation to occur [28]. Inflammation is associated with the production of reactive oxygen species (ROS), which activate MUC1-C by inducing the formation of MUC1-C homodimers. ROS-induced activation of the Cys residues confers the formation of homodimeric MUC1-C complexes and heterodimeric complexes with other proteins, such as MYC [29]. MUC1-C can regulate intracellular ROS levels through the induction of NADPH and glutathione production. Additionally, MUC1-C stimulates the inflammatory NF-κB/p65 pathway, promoting the EMT transcriptional repressor zinc-finger E-box-binding homeobox 1 (ZEB1) and causing epithelium metamorphosis [30]. Notably, MUC1 has anti-inflammatory effects and reduces inflammation when it targets DCs.

Additional studies have shown that MUC1 overexpression by cancer cells is associated with protection from natural killer cells (NK cells), cytotoxic T cell-mediated lysis, and TRAIL- and Fas ligand-induced apoptosis, suggesting that MUC1 contributes to immune evasion in aggressive cancer [31,32].

Moreover, MUC1 increases PD-L1 expression directly at the transcriptional level, induces primary healthy monocytes to differentiate into macrophages with a tumor-associated macrophage M2-like (TAM2) phenotype, and decreases MCP1, IFN-gamma, and GM-CSF expression [33,34,35].

## 5. Mucins: Tumorigenesis and Metastasis

Under normal circumstances, epithelial cells show a distinct polarity, with RTKs located on their basolateral membranes and mucins on the apical interface. Cell–cell and cell–matrix interactions are necessary to preserve the integrity of the epithelium. Epithelial cells lose their tight connections because of persistent inflammation and stress-induced signaling, which trigger pathways for reversible repair and growth [36,37]. The lack of polarity causes mucins to disperse on both sides of the epithelial membrane, resulting in an aberrant mucin interactome with membrane-associated junctional complex members and homo- and heterodimers of RTKs. The mesenchymal phenotype becomes more enriched as a result of this event. It stimulates the inflammatory tissue’s survival programming, which causes mucin hypersecretion, as shown in goblet cell hyperplasia [38]. Research indicates that transmembrane mucins, such as MUC1 and MUC4, can interfere with adherens and tight junctions during oncogenesis due to their distinct domains. These events promote the differentiation, repair, and survival pathways. Instead, malignant cells use mucin pathways to communicate constitutive growth and proliferation, resulting in tumorigenesis. However, the role of secreted mucins in tumorigenic onset is still elusive [39]. Most cancer-related deaths are caused by metastasis, yet little is known about the molecular actors and how they contribute to this cascade. Malignant cells must go through a complicated series of processes to colonize distant organs. These include invading the surrounding stroma to reach blood vessels, surviving in the bloodstream, extravasating through transendothelial migration, homing to the secondary site, and surviving and proliferating in the metastatic organs [40]. Glycans on the extracellular domains of mucins appear to mediate interactions with immune cells and the tissue environment in the circulation, transferring signals from the outside in [41,42]. Endothelial cells produce E-selectin during inflammation and cancer progression. This protein interacts with the sialylated glycans (SLeX, SLeA) of tumor cell mucins, beginning the docking process of tumor cells on endothelial cells [43]. The rolling process increases integrin expression and causes significant contact with intercellular adhesion molecule-1 (ICAM-1) on endothelial cells, leading to extravasation. It has been demonstrated that integrin expression and interaction with ICAM-1 are upregulated by several mucins, including MUC1, which increases the invasiveness and extravasation capacity of tumor cells [30]. Nevertheless, the real connection between mucins and metastasis is still unknown. Anyway, circulating levels of mucins have been shown to be correlated with cancer clinical stage in multiple investigations.

Anoikis is a type of programmed cell death occurring in anchorage-dependent cells when they detach from the surrounding extracellular matrix. Anoikis resistance represents an emerging hallmark of the metastatic potential of cancer cells since it provides cells with an advantage of anchorage-independent survival during metastatic spread. Zhao et al. showed that MUC1 overexpression prevented the initiation of anoikis in epithelial cancer cells in response to the loss of adhesion. In particular, MUC1 expression not only inhibited integrin-mediated anoikis initiation by preventing integrin activation but also prevented death receptor-mediated anoikis initiation by preventing ligation of the cell surface death receptors with their ligands in response to the loss of cell adhesion. Such effects were largely due to the elongated and highly glycosylated extracellular domain of MUC1 [44].

The role of MUC1 extracellular domain *O*-glycosylation on anoikis resistance was investigated in a later study that demonstrated how inhibition of *O*-glycosylation by suppression of Core 1Gal-transferase (C1GT, T-synthase) expression significantly increased anoikis in MUC1-positive cancer cells. C1GT is a glycosyltransferase involved in the synthesis of *O*-linked mucin-type glycans [45]. C1GT leads to the formation of the Core 1 structure (Galβ1,3GalNAcα-, T or TF antigen) that is the predominant structure of MUC1 O-glycosylation in epithelial cancer cells [46,47,48].

Finally, Bose et al. showed that the use of an anti-MUC1 antibody inhibited ligand-dependent and -independent mechanisms of anoikis resistance and reduced pancreatic cancer cell survival and invasion in vitro and in a xenograft model [49].

## 6. The Role of MUC1 in Chemotherapy Resistance

Poor response to therapy due to the development of resistance in tumors remains a serious therapeutic problem, contributing to overall poor patient prognosis. Chemotherapy resistance is regulated by different mechanisms, including expression of drug pumps, reduced drug intake, reduced susceptibility to apoptosis, increased ability to repair DNA damage, expression of detoxification systems, epithelial–mesenchymal transition, and metabolic reprogramming. Many studies have shown that abnormal mucin expression can induce chemoresistance in some malignancies. In pancreatic tumors, the hypoxic microenvironment stabilizes HIF-1α, the master regulator of glucose metabolism, and causes glucose dependency in cancer cells [50]. Previous research has shown that the cytoplasmic tail of MUC1 physically interacts with and blocks the proteasomal degradation of HIF-1α in pancreatic cancer cell lines and animal models, and higher expression of MUC1 was observed in gemcitabine-resistant cells [51].

MUC1 expression is a key predictor of RCC response to a variety of anticancer drugs with unique mechanisms of action. Given the unrelated chemical structures of the studied drugs, MUC1-overexpressing cells displayed a multidrug-resistant profile, indicating the activation of drug efflux transporters. Many findings suggest that the expression of ATP-binding cassette (ABC) transporters, especially multidrug-resistant protein 1 (MDR1, also known as P-glycoprotein), which is encoded by the ABC subfamily B member 1 (ABCB1) gene, confers resistance to both cytotoxic and targeted therapies [52,53]. The multidrug resistance phenotype in cancer cells results from increased removal of anticancer drugs, leading to reduced drug accumulation within cells. This is mainly driven by the overexpression of ABC transporters and activation of the PI3K/Akt and Erk1/2 pathways. Notably, PI3K/Akt activation regulates ABCC1 gene expression in prostate cancer cells [54,55]. Moreover, overexpression of MUC1 in cancer cells hyperactivates both Erk1/2 and PI3K pathways, contributing to drug resistance in MUC1-overexpressing prostate cancer cells.

MUC1 was previously shown to give chemoresistance in pancreatic cancer cells by upregulating multidrug resistance protein-1 (MRP1) [56]. ABCB1 was identified as a key mediator of MUC1-dependent chemoresistance in cervical cancer and pulmonary mucoepidermoid lung carcinoma. In addition, MUC1 promotes the activation and nuclear distribution of EGFR. Together, EGFR and MUC1 upregulate ABCB1, which contributes to chemoresistance development [57]. Ham et al. evaluated the role of MUC1-C in promoting stemness and paclitaxel (PTX) resistance in human NSCLC A549 cells [58]. Recent cancer treatment research focuses on lowering CSC populations, which can cause drug resistance. MUC1-C is one of the targets for reducing PTX resistance and stemness in cancer treatment. Overall, combining a MUC1-C inhibitor with an anticancer drug may have a synergistic impact in cancer patients with PTX-resistant tumors.

## 7. MUC1 in Human Carcinoma

Nearly all human cancers, including over 90% of breast, pancreatic, ovarian, bladder, lung, and kidney cancers, and multiple myeloma, exhibit aberrant overexpression of MUC1 [59,60,61]. MUC1 is significantly expressed in 77% of primary lung malignancies and 70% of human colon tumors, and tumor-associated MUC1 differs from normal cell-expressed MUC1 in terms of both cellular distribution and biochemical characteristics [62,63,64,65]. Tumor-related MUC1 exhibits certain features, such as loss of apical localization toward a peripheral one and aberrant glycosylation that might cause the creation of tumor-related antigen epitopes [66,67,68]. These tumor-associated alterations also impact multiple pathways involved in the advancement of cancers [2]. Moreover, these include pharmacodynamic inhibitors and their anti- and pro-inflammatory effects within different infection-caused malignancies via an immunomodulatory switch [69,70]. However, MUC1 also causes radio- and chemoresistance during cancer treatment and promotes tumor invasion and migration [64]. Although the exact role of MUC1 in tumor progression is unknown, there is evidence to suggest that it may play a role in several oncogenic pathways, including cell adhesion, differentiation, apoptosis, proliferation, and angiogenesis [71].

### 7.1. MUC1 in Breast Cancer

Breast cancer (BRCA), a malignant epithelial tumor of ductal or lobular origin, represents a major cause of cancer-associated mortality in females even with significant improvements in therapy. Numerous researchers over the past three decades have looked into an immunotherapeutic method of targeting BRCA as tumor immunotherapy. The investigation of MUC1 as a potential target for BRCA immunotherapy is still ongoing. MUC1 expression partially increases tumor invasion and metastasis and promotes the creation of tumor blood vessels. Vascular endothelial growth factor (VEGF) and MUC1 expression are strongly correlated with BRCA mutations, and MUC1 expression has been demonstrated to increase blood vessel formation in BRCA patients, both in vitro and in vivo [72,73]. A different mechanism of MUC1 is associated with the regulation of tumor cell proliferation and apoptosis, which is connected to regulating multiple tumor cell proliferation/apoptosis pathways. When MUC1-C, but not MUC1-N, is overexpressed in BRCA, polarity loss and transformation of epithelial cells generally result in overexpression of MUC1, which can subsequently increase resistance to stress-induced apoptosis while increasing transformation [74,75]. Three primary strategies to target MUC1 in relation to BRCA have been described [76]. MUC1′s glycopeptide epitope has long been recognized to generate both cellular and humoral adaptive responses. MUC1 has an impact on the phenotypes and functions of immune cells in the tumor microenvironment (TME). The last strategy is MUC1-C, a small protein that bridges a membrane [71]. Current data indicate that MUC1 is among the most significant targets for BRCA immunotherapy, and additional research on MUC1 will probably be crucial to unlock a “door of hope” for anti-BRCA immunotherapy.

Triple-negative breast cancer (TNBC) accounts for about 10–15% of all BRCA, and patients with this aggressive form of tumor present an increased risk of recurrence and metastatic disease compared to other BRCA subtypes [77]. This denomination refers to the fact that the cancer cells are estrogen receptor/progesterone receptor/HER2 negative. MUC1 drives the reprogramming and dedifferentiation of TNBC cells and regulates metabolic reprogramming, in particular glutamine metabolism, by increasing the transcription of genes regulating glutamine uptake, aminotransferases, and glutaminolysis [78,79].

More recent studies have investigated the role of MUC1 in the mechanisms of tumor immune evasion. MUC1-C activates the inflammatory interferon (IFN)-gamma-driven JAK1-STAT1-IRF1 pathway, which plays an important roles in immunosuppression, and it is associated with the depletion and dysfunction of CD8+ T cells in the TNBC tumor microenvironment [80].

In this scenario, a recent study showed that MUC1-C is a druggable target to sensitize TNBC cells to carboplatin and olaparib. Targeting MUC1-C inhibits the chronic activation of the type I IFN pathway that stimulates the cGAS/STING axis, inducing the IFN-related DNA damage resistance gene signature [81].

### 7.2. MUC1 in Colorectal Cancer

Colorectal cancer represents one of the most prevalent cancers and the second leading cause of cancer-related death worldwide. MUC1 is significantly expressed in colon cancer and has been associated with adverse outcomes. In particular, the progression of colorectal cancer can be promoted by pro-inflammatory cytokines and tumor-infiltrating myeloid cells [82]. Epithelial cells in the gastrointestinal system frequently produce many mucins, even if an organ-specific pattern of mucin predominance may exist. For instance, MUC2 is often found in the goblet cells of the mucosa of the small and large bowel, whereas MUC1 is expressed on the apical surface of most epithelial cells [83]. Notably, mucin expression may decrease or lose its organ specificity during neoplastic transformation and/or progression, whereas novel mucins may be abnormally expressed [84]. It has been demonstrated that MUC1 overexpression in epithelial tumor cells is essential to the transformation process, but little is known about MUC1′s function in immune cells throughout the development of cancer and in regulating the interaction between tumor cells and the immune system. Sheng et al. demonstrated that in the azoxymethane (AOM)/dextran sulfate sodium (DSS) animal model of colitis-associated cancer (CAC), MUC1 acts as a neoplastic factor that controls macrophage infiltration and function and promotes a tumor-permissive environment that affects tumor initiation and progression to invasive cancer [85]. MUC1 has a significant role in immune cell signaling, which encourages the development of cancer linked to chronic inflammation. These novel findings add to MUC1′s potential as a therapeutic target by showing that MUC1 in innate immune cells promotes the development of cancer, at least in colorectal cancer.

In a recent study, Morimoto et al. showed, in a poorly differentiated colon carcinoma cell line, that MUC1-C represses MICA and MICB expression in cancer cells and inhibits the cytotoxic activity of NK cells [86].

### 7.3. MUC1 in Prostate Cancer

Treatments for castration-resistant prostate cancer (CRPC) that target the androgen receptor (AR) axis, such as abiraterone and enzalutamide, are increasing. However, individuals with CRPC frequently advance to a more aggressive variant with neuroendocrine (neuroendocrine prostate cancer, NEPC) characteristics and become resistant to AR-targeted treatment. Treatment-related NEPC (t-NEPC) has a poor prognosis and few treatment choices, and it is extremely aggressive. Research using human NEPC cell models has shown that MUC1-C inhibits the AR axis [87,88]. Prolonged MUC1-C activation is linked, as we described, to carcinogenesis and lineage plasticity. Research using human NEPC cell models has shown that MUC1-C promotes the IFN and the NOTCH signaling in pancreatic ductal carcinoma (PDAC-NE) cells and inhibits the AR axis [89]. MUC1-C, via direct interactions with MYC, increases the synthesis of the BRN2 neural transcription factor (TF) and other NE phenotypic effectors, including ASCL1 [90]. To support the NEPC cancer stem cell (CSC) state, MUC1-C additionally activates the NOTCH1 stemness TF. It has been suggested that one of the variables influencing intra-tumor heterogeneity is CSCs [91]. On the other hand, new research has demonstrated that a tumor cell’s ability to look like a stem cell is flexible. This perspective implies that stemness features can be acquired through interactions with the tumor microenvironment (TME) and/or (epi)genetic alteration. By establishing a differentiation hierarchy that results in a variety of distinct cell types existing inside the tumor, CSCs produce cellular heterogeneity [92]. NEPC self-renewal, tumorigenicity, and treatment resistance are all inhibited by targeting MUC1-C [93,94]. Anti-MUC1 drugs are now undergoing preclinical and clinical trials. Since MUC1-C is a target for the treatment of these aggressive malignancies, anti-NE carcinomas, such as Merkel cell carcinoma (MCC) and small-cell lung cancer (SCLC), are additionally dependent on it.

### 7.4. MUC1 in Lung Cancer

The most prevalent kind of cancer diagnosed worldwide is lung cancer, which is also the leading cause of cancer-related deaths and is typically linked to unfavorable clinical outcomes. Immune cells become inactive upon binding to inhibitory receptors (immune checkpoints), such as programmed death 1 (PD-1) and Cytotoxic T Lymphocyte-Associated Protein 4 (CTLA4) [95,96]. Immunotherapy based on immune checkpoint inhibitors is a milestone in the treatment of systemic cancers. Indeed, the treatment landscape for non-small-cell lung cancer (NSCLC) has improved with the blocking of immune checkpoints such as PD-L1 and PD-1. A growing body of research indicates that MUC1-C controls a wide range of genes, including PD-L1, which encourages NSCLC cell escape and reduces the impact of immune cells [97]. Consequently, PD-L1 is downregulated after targeting MUC1-C [98].

MUC1 acts also as a prognostic factor in patients with NSCLC. In particular, Kaira et al. showed how depolarized MUC1 expression was associated with poor clinical outcomes [99]. Higher expression of MUC1 had already been unveiled in lung squamous cell carcinoma and adenocarcinoma. Patients with squamous carcinoma and lung adenocarcinoma had higher percentages of elevated MUC1 expression (86.3 and 39.1%, respectively) in prior research involving 178 patients with stage IB NSCLC [100]. These results point to a significant involvement of MUC1 in the development of lung adenocarcinoma.

### 7.5. MUC1 in Ovarian Cancer

Ovarian cancer is regarded as one of the most aggressive gynecological cancers due to its early ability to metastasize [101]. Ovarian cancer cells’ metastasis, progression, and treatment resistance might also be attributed to MUC1 [102]. The positive expression rate of MUC1 was found to be 95.0% (57/60) in research including 60 primary ovarian cancer tissue specimens that were paraffin-embedded and sectioned. The elevated expression rate of MUC1 is linked with the stage of the tumor and the amount of remaining tumor tissue after surgery [103]. According to certain theories, MUC1 contributes to the growth of ovarian cancers and the unfavorable prognosis of patients.

### 7.6. MUC1 in Bladder Cancer

Globally, there are approximately 573,000 new cases of bladder cancer (BC) every year, and the disease accounts for about 213,000 deaths, making it the ninth most common [104,105]. In healthy urinary epithelium, MUC1 helps to preserve mucosal integrity and prevents bacterial invasion. However, aberrant expression of MUC1 is linked to the progression and metastasis of BC [106]. In benign bladder urothelium, it was shown to be either confined to umbrella cells, as reported in most cases, or to form a sheath over transitional epithelium, as observed in a small number of cases, according to immunohistochemical investigations using the HMFG2 antibody. Additionally, under some circumstances, the superficial layer of the bladder epithelium has strong MUC1 expression [102]. According to Elazeez et al., MUC1 was detected in 74% of papillary transitional cell carcinomas. Additionally, it was also reported that the higher the nuclear grading, the higher the MUC1 expression: 37.5% of grade 1 cases, 75% of grade 2 cases, and 88.9% of grade 3 instances. This difference in expression was statistically significant (*p* = 0.01) [107]. Similar findings were made by another study, which recognized an increasing expression in benign bladder illness, bladder tumors, and normal bladder mucosa [108]. It has been proposed that MUC1 has a role in bladder cancer development and treatment resistance [109]. Because MUC1 has a high diagnostic accuracy for bladder cancer, it may be a useful clinical marker and targeted treatment molecule.

### 7.7. MUC1 in Pancreatic Cancer

The poorest prognosis and the fastest progression characterize pancreatic adenocarcinomas. Missing the best timing for surgery results in poor treatment outcomes for the majority of patients [110]. According to a prior study, aberrant MUC1 overexpression is seen in over 60% of pancreatic cancers, and this finding is associated with a bad prognosis for the patients. According to certain theories, MUC1 has a function in the onset and spread of pancreatic cancer and may be an important marker for detection [111]. Furthermore, MUC1 activity could enhance glucose metabolism in cancer cells [10]. A low-MUC1 expression group was associated with a longer median survival time (39.7 months) than a high-MUC1 expression group (13.4 months). These results suggested that MUC1 may be a key target molecule to assess and enhance patient prognosis, as it is linked to a poor prognosis and short survival time in pancreatic cancer patients [112].

## 8. MUC1 Expression in Kidney Cancer

Clear-cell renal cell carcinoma (ccRCC) represents the most common histology of renal cell carcinoma (RCC), followed by papillary RCC and chromophobe RCC. In the US, 79,000 new cases of ccRCC were reported in 2022, and around 13,920 individuals died of this disease [113]. Studies performed using tissue microarrays have shown that MUC1 is a useful diagnostic and prognostic marker in renal tumor pathology and that MUC1 is significantly more expressed in metastatic RCC compared to primary tumors. Moreover, MUC1 was particularly overexpressed in sarcomatoid components, corresponding to the areas in which the cancer cells had undergone epithelial-mesenchymal transition [114,115].

Recent research has established that RCC is essentially a metabolic disease, even though the pathophysiology of the disease is still not entirely understood. Numerous studies have demonstrated that changes in metabolic pathways have a part in the development of RCC and that a significant amount of altered genes in this disease are essential for regulating the metabolic activities of cells [116,117].

A specific metabolic profile of ccRCC has been identified considering that it is characterized by significant metabolic reprogramming. ccRCC displays a substantial accumulation of polyunsaturated fatty acids, decreased mitochondrial activity, and a redirection of the glucose metabolism toward the pentose phosphate pathway (PPP) [118,119,120,121,122,123]. Differentiating across cancer subtypes according to the molecular traits that drive an aggressive phenotype is a key objective in current research as it can aid in the prediction of clinical outcomes.

A recent study investigated the role of MUC1 in ccRCC [124]. A metabolomic analysis in ccRCC with different MUC1 expression levels (MUC1 high vs. MUC1 low) and normal renal tissues showed that MUC1-high (MUC1H) ccRCCs exhibited a particular metabolic rewiring involving glucose and lipid pathways. Elevated glucose levels were associated with an increase in the upstream glycolytic intermediates and a decrease in the downstream metabolites in MUC1H tumors. These results, along with the high expression levels of TKT, PPP intermediates, and G6PDH, point to a higher rerouting of the sugar metabolism toward this pathway to support redox homeostasis and anabolic processes, particularly in MUC1H ccRCC Alteration of lipid metabolism represents another important hallmark of ccRCC. It has been demonstrated that ccRCC is characterized by a high buildup of polyunsaturated fatty acids (PUFAs) and overexpression of stearoyl-CoA desaturase-1 (∆-9-desaturase; SCD1) and fatty acid elongase 2 and 5 (ELOVL2 and ELOVL5) [125]. Moreover, it was found that these alterations were more evident in MUC1H ccRCC compared to MUC1-low (MUC1L) tumors and normal tissues. Citrate and succinate levels were considerably higher in MUC1H tumors than in MUC1L ccRCC and normal tissue, but fumarate and malate levels were significantly lower in cancer tissue. These amounts of Krebs cycle metabolites suggested a metabolic profile that might be associated with improved glutaminolysis. Almost all amino acids were dramatically decreased in tumor tissue, except for glutamate, glutamine, and cysteine, which were significantly enhanced in MUC1H tumors.

MUC1 modulates chronic inflammation, but little is known about how it controls the tumor microenvironment (TME), particularly in ccRCC [124].

Gene set enrichment analysis (GSEA) was used to examine the changes in the gene expression in ccRCC MUC1H vs. MUC1L. MUC1H ccRCCs show higher expression of genes involved in immune cell infiltration, hypoxia, angiogenesis, complement system activation, and epithelial–mesenchymal transition (EMT) [126]. The GSEA data indicated that MUC1 was also involved in other aspects of tumor biology, such as regulation of immune cell infiltration and angiogenesis induction. Next, the tissue expression of PTX3 and the complement system’s activation in tumor samples were evaluated. Vascular density was assessed by using CD31 immunostaining, and MUC1H ccRCC samples exhibited a considerable increase compared to MUC1L [127]. Besides protein synthesis, tryptophan (TRP) can be converted into kynurenine (KYN) by two different enzymes: tryptophan 2,3-dioxygenase (TDO) and indoleamine 2,3-dioxygenase (IDO1). Upon its interaction with aryl hydrocarbon receptor (AhR), KYN plays a tumorigenic role in cancer and immune cells together with TRP depletion in the TME. IDO1+ macrophages sustained enhanced accumulation of KYN in ccRCC, and activation of the KYN pathway increased renal cancer cells’ survival, migration, and chemoresistance [128]. MUC1H ccRCC showed a greater infiltration of M2 tumor-associated macrophages CD68+CD163+ (TAMs) that were able to produce KYN by IDO1. In addition, the infiltration of CD8+ and CD4+ T cells, as well as PD-L1 expression, was examined in the ccRCC microenvironment. Remarkably, MUC1H tumors showed a significant reduction in CD8+ T cells, whereas CD4+ T cells accumulated. PD-L1 expression was reduced in MUC1H tumors [113].

Pentraxin-3 (PTX3) is an acute-phase reaction protein that has been demonstrated to be able to activate the classical complement cascade via C1q in the TME. Indeed, PTX3 has been found to colocalize with C1q. In ccRCC, it has been suggested that PTX3 controls carcinogenesis either directly or indirectly. Released anaphylatoxins C3a and C5a affect the immunoflogosis in the TME by activating mast cells, myeloid-derived suppressor cells (MDSCs), and TAM2 [129,130]. Increased deposition of C1q and expression of proangiogenic receptors (C3aR and C5aR) have been demonstrated in MUC1H. Nonetheless, the assembly of membrane attack complex (MAC) C5b-9 was limited by enhanced expression of CD59 (complement inhibitor) [131].

CA15-3 is the soluble form of MUC1. High blood levels of CA15-3 have been linked in the past to an advanced stage of RCC. Grankvist et al. found that up to 30% of individuals with high-grade and high-stage RCC had elevated levels of CA15-3. These findings were supported by a more recent study that demonstrated a shorter cancer-specific survival (CSS) and progression-free survival (PFS) for clear-cell and non-clear-cell RCC patients with increased CA15-3 levels [132]. Preoperative levels of CA15-3 were measured (normal range: 0–25 U/mL) in both patients undergoing nephrectomy for RCC and healthy volunteers. Compared to healthy individuals, ccRCC patients had higher serum levels of CA15-3. Importantly, higher levels were measured in patients with higher nuclear grade (G3 and G4) and lymph node and visceral metastasis at the time of the diagnosis. In addition, worse CSS and PFS were associated with higher blood levels of CA 15-3 according to Kaplan–Meier survival curves [133]. Intriguingly, serum levels decreased one month after surgery for RCC. High levels of CA15-3, together with the presence of visceral and nodal metastases, nuclear grade, and advanced stage were independent factors for CSS and PFS, according to multivariable analysis [109]. By stimulating the complement system’s classical route and controlling the immune infiltration, MUC1 regulates immunoflogosis in the ccRCC microenvironment and encourages the development of an immunological-silent microenvironment. MUC1-expressing ccRCC has specific characteristics such as modified metabolism, elevated microvascular density, elevated TAM2, reduced immune infiltration, and decreased PD-L1 expression [134,135]. Therefore, these tumors might better respond to antiangiogenic treatments than to immune checkpoint inhibitors (ICIs) because of their specific phenotype.

The recent development of methods for detecting, analyzing, and monitoring circulating tumor cells (CTCs) in different body fluids has contributed to a better characterization of tumors and to an improved classification of patients according to different risk groups [136].

The well-known molecular heterogeneity of renal CTCs implies the need to use combined markers and methodologies to identify these cells. A recent study showed that MUC1 is a surface marker that can be effectively used to identify renal CTCs in the blood [137]. In addition, its expression in a greater number of CTCs was predictive of the development of lung metastases.

Many studies have suggested that stem cell-like CTCs could have a role in promoting the evolution and progression of different tumors [91]. The identification and characterization of cancer stem cells (CSCs) in renal cell carcinoma is still under investigation [138,139]. Galleggiante et al. described and characterized a population of resident CD133+/CD24+ cancer cells in patients with clear-cell RCC [140]. In addition, Varna et al. identified a larger number of CD133/CXCR4-coexpressing cells in perinecrotic versus perivascular areas in RCC tissue [141].

The use of CD105 as a renal CSC marker was questioned in many studies that showed how other putative subpopulations of cells with CSC-like properties are CD105- [142,143]. Canis et al. showed that stable transfection of CD133 in the human embryonic kidney 293 (HEK293) cell line induced tumor-initiating properties in these cells. Moreover, HEK293 CD133high transfectants, when injected into SCID mice, generated tumors with at least a 1000-fold increased frequency as compared with CD133low cells [144]. In this scenario, it has been shown that MUC1 can promote cancer stemness and could be used to identify circulating stem cell-like tumor cells [145,146,147,148,149,150].

## 9. Conclusions

The two subunits of the heavily glycosylated protein MUC1 have distinct functions in both healthy and pathological conditions. Under normal circumstances, the N-terminus acts as a barrier to shield cells from bacterial infections and harmful stimuli. The C-terminus is found within cells and plays an important role in various signaling pathways that influence and control tumor development, migration, invasion, apoptosis, and survival [151,152,153,154,155]. MUC1 acts as a co-transcriptional activator and has an important influence on metabolism. Under pathological circumstances, MUC1 acts in a pro-inflammatory way, producing positive conditions for tumor growth. Undoubtedly, MUC1 causes inflammation to turn into cancer, increases treatment resistance, enhances tumor spread, and is involved in cancer progression. MUC1 overexpression has been detected in a variety of epithelial cancer types, including RCC, making it a useful marker for prognosis and diagnosis in clinical settings.
